# PxFquery: A Bioinformatics Tool for Large Language Model-Assisted Functional Analysis of Large-Scale Perturbation Signatures

**DOI:** 10.3390/genes17091014

**Published:** 2026-08-27

**Authors:** Jun Cao, Xiaoyue Wang

**Affiliations:** State Key Laboratory of Complex, Severe, and Rare Diseases, Center for Bioinformatics, National Infrastructures for Translational Medicine, Institute of Clinical Medicine, Peking Union Medical College Hospital, Chinese Academy of Medical Sciences and Peking Union Medical College, Beijing 100730, China; caoduduyzy@gmail.com

**Keywords:** perturbation signatures, functional analysis, large language models, Connectivity Map, LINCS, functional genomics

## Abstract

**Background/Objectives**: Genetic and chemical perturbation experiments provide a systematic approach to investigate cellular responses. Large-scale resources such as Connectivity Map (CMap) contain extensive transcriptomic perturbation signatures that support functional interpretation and perturbation retrieval. However, existing access to these resources mainly relies on structured inputs, making it challenging to connect natural-language perturbation questions with experimental evidence. **Methods**: To address this challenge, we developed PxFquery, an evidence-grounded tool that enables natural-language exploration of large-scale perturbation resources. PxFquery uses an LLM-assisted workflow to interpret biological questions and organize responses based on retrieved perturbation evidence. It converts over 500,000 CMap/LINCS perturbation signatures into a compact functional response space and supports bidirectional perturbation-function queries. **Results**: Despite the sparse perturbation coverage of CMap (5.9%), PxFquery integrated related perturbation evidence and improved access to perturbation resources. Across evaluated genetic and chemical perturbation-to-function and function-to-perturbation queries, PxFquery outputs showed closer agreement with experimental reference rankings than direct and PubMed-augmented LLM approaches (paired Wilcoxon tests; *p* < 0.05). Functional response representation reduced storage requirements to 0.068–0.24% of the original resources and enabled lightweight deployment through a Python package (v0.5.29), website, and AI workflow interfaces. **Conclusions**: PxFquery provides a natural-language interface for exploring large-scale perturbation resources while maintaining connections to experimental evidence. It lowers the barrier to accessing these resources and enables their integration into diverse AI workflows.

## 1. Introduction

Understanding how genetic and chemical changes affect cellular states is a fundamental goal in biology. Researchers often address this question by performing genetic or chemical perturbation experiments and measuring the resulting molecular changes. These experiments have generated large-scale transcriptomic perturbation signatures that record cellular responses under different perturbation conditions. The Connectivity Map (CMap), later expanded through the Library of Integrated Network-based Cellular Signatures (LINCS) L1000 program, is a major resource that organizes such perturbation signatures [1,2]. By collecting millions of perturbation profiles, CMap/LINCS provides experimental evidence for comparing perturbation effects and interpreting cellular responses [2,3].

A key step in using perturbation resources is to translate molecular responses into functional evidence that can answer biological questions. Existing approaches mainly address this task in two directions. In the perturbation-to-function direction, transcriptomic changes from measured perturbation signatures are mapped to predefined functional programs through gene-set-based approaches, including over-representation analysis (ORA) and gene set enrichment analysis (GSEA) [4,5]. The Molecular Signatures Database (MSigDB) and its Hallmark gene sets provide widely used functional references for this type of interpretation, and tools such as clusterProfiler and GSEApy facilitate routine enrichment analysis [4,5,6,7]. In the function-to-perturbation direction, researchers can use functional gene sets or molecular features to identify perturbations with similar or opposite response patterns. L1000CDS2 and L1000FWD implemented this signature-based retrieval strategy for LINCS L1000 data and supported perturbation mechanism analysis and drug repositioning studies [8,9,10,11]. Beyond these analysis methods, platforms such as LINCS Canvas Browser, CLUE, SigCom LINCS, and iLINCS provide structured interfaces for accessing perturbation signatures through gene sets or metadata-based queries [2,12,13,14].

However, directly connecting large-scale perturbation resources with biological questions expressed in natural language remains challenging. The first challenge is how to ground natural-language perturbation queries in experimental evidence. Recent studies such as PerturbQA and SUMMER have shown that large language models (LLMs) can interpret perturbation-related questions by integrating experimental information and domain knowledge [15]. However, these approaches focus on specific single-cell perturbation experiments rather than accessing reusable perturbation resources across diverse cellular contexts, such as CMap/LINCS. The second challenge is that large-scale perturbation resources have limited experimental coverage, as only a small fraction of possible perturbation-cell combinations have been measured. Studies such as GEARS, GenePert, and LangPert have explored the use of related information from measured records to predict perturbation responses beyond directly measured conditions [16,17,18]. However, these approaches mainly focus on predicting perturbation-associated molecular responses rather than querying functional consequences from large-scale perturbation resources. These challenges limit researchers from directly obtaining evidence-supported perturbation insights from biological questions.

Here, we present PxFquery, a bioinformatics tool that connects natural-language biological questions with evidence-grounded perturbation resources from CMap/LINCS. PxFquery converts more than 500,000 CMap/LINCS perturbation signatures into a compact functional response space and supports two complementary query directions: perturbation-to-function and function-to-perturbation. To address the challenge of natural-language access, PxFquery uses an LLM-assisted workflow to interpret queries, organize evidence, and generate responses. To expand evidence availability beyond directly measured conditions, PxFquery integrates cell-line relationships, chemical structural similarity, and gene embedding similarity to identify related perturbation evidence. We evaluated PxFquery across genetic and chemical perturbation-to-function and function-to-perturbation queries, showing closer agreement with experimental reference rankings than LLM-based approaches. PxFquery is available as a lightweight Python package, web application, and MCP interface for exploring large-scale perturbation resources.

## 2. Materials and Methods

### 2.1. Data Resources

#### 2.1.1. CMap/LINCS Perturbation Signatures

We obtained CMap/LINCS Level 5 perturbation signatures from the CLUE data release platform and used them as the source transcriptional response resource [2]. Here, chemical perturbation refers to treatment with a compound, including drugs and other small molecules. The analysis included 201,014 cp (compound) signatures, 189,365 sh (short hairpin RNA; shRNA) signatures, and 132,464 xpr (CRISPR) signatures (Table S1). Each signature was associated with a measured cell line and perturbation identifier, providing the experimental context used for functional scoring and query matching.

#### 2.1.2. Functional Gene Sets

Functional responses were represented in a 91-gene-set functional program space. This collection included 50 MSigDB Hallmark gene sets (v2024.1.Hs) and 41 Curated Cancer Cell Atlas meta-programs (3CA MPs) (Table S2) [6,19]. The MSigDB Hallmark collection provided broad, canonical biological programs, whereas the 3CA MPs added patient-tumor-derived cancer-cell programs from single-cell tumor atlases. Combining these two collections produced a 91-program space that covered both general cellular processes and cancer-context transcriptional states. The same 91 gene sets were used for cp (compound), sh (shRNA), and xpr (CRISPR) signatures, allowing all perturbation classes to be scored in a shared functional space.

#### 2.1.3. Annotation Resources

External annotation resources were used to standardize entity names or terms in natural-language queries before linking them to CMap/LINCS records. Cell-line names were standardized using Cellosaurus, which provided cell-line aliases together with lineage, disease, and subtype information [20]. Gene names were standardized using GENCODE v49 and CMap gene metadata [2,21]. Compound names were standardized using CMap/LINCS compound metadata and PubChem chemical identifiers and molecular structures, connecting CMap names, BRD identifiers, and compound aliases [2,22].

### 2.2. Precomputed Resource Construction

#### 2.2.1. Functional Score Matrices

For each CMap/LINCS Level 5 signature, genes were ranked by their Level 5 response values and analyzed against the 91-gene-set collection using GSEApy v1.1.13 for preranked gene set enrichment analysis with 1000 permutations [4,7]. The 5th–95th percentile range of NES values was −1.935 to 1.832 (Figure S4).

For signature i and gene set j, the functional score was defined [23,24] as $S_{ij}=NES_{ij}\cdot \left[-\log_{10}(FDR_{ij}+\varepsilon )\right]$, where $NES_{ij}$ is the normalized enrichment score (NES), $FDR_{ij}$ is the false discovery rate (FDR) q value. The FDR-derived weighting term is incorporated to refine the representation of functional enrichment signals according to their statistical support, particularly among signals with similar enrichment magnitudes (Figure S5). *ε* is a small positive numerical stability constant set to 10^−10^ to avoid taking the logarithm of zero. The robustness of this *ε* value was confirmed by sensitivity analysis using alternative *ε* values (Figure S6).

Scores from all signatures were assembled into cp (compound), sh (shRNA), and xpr (CRISPR) signature-by-program matrices that shared the same 91 functional-program columns. For use in the PxFquery package (v0.5.29), the precomputed score matrices were stored in a compact numeric format by rounding scores to two decimal places, storing values in half precision, and setting scores with absolute values below 0.1 to zero. These functional score matrices were used as internal evidence scores in PxFquery to organize perturbation-associated functional evidence and support natural language-based responses.

#### 2.2.2. Cell-Line Hierarchy

In coordination with the standardized cell-line annotations described above, cell lines in CMap were organized into a hierarchical cell-line tree defined by lineage, disease, and subtype. The tree retained exact cell-line identities while providing progressively broader cellular contexts. When a matching cell line is available, PxFquery prioritizes exact cell-line evidence. When exact cell-line evidence is unavailable, the tree is used to locate related cellular contexts within the same disease, subtype, or lineage, supporting cellular-context retrieval and matching during query processing.

#### 2.2.3. Compound and Gene Similarity References

Similarity references were constructed to retrieve related perturbation evidence when direct measured records were unavailable in the queried cellular context. Based on the standardized compound structures, Morgan fingerprints with radius 2 and 2048 bits were generated using RDKit v2026.03.1, and pairwise Tanimoto similarities were calculated [25,26]. For each compound, the 50 nearest structural neighbors were retained, and neighbors with Tanimoto similarity of at least 0.30 were used during query matching as related compound evidence. Threshold robustness was tested by varying the Tanimoto similarity cutoff from 0.20 to 0.40 (Figure S10).

Gene similarity references were constructed from GenePT model-3 embeddings to identify matrix-backed genetic perturbations related to a queried gene [27]. Embeddings were L2-normalized and compared by cosine similarity against genetic perturbation candidates represented in the score matrices. For each query gene, the 50 nearest matrix-backed genes were retained, and neighbors with cosine similarity of at least 0.50 were used during query matching as related genetic evidence. Threshold robustness was tested by varying the cosine similarity cutoff from 0.40 to 0.60 (Figure S10).

### 2.3. PxFquery Analysis Workflow

#### 2.3.1. Perturbation Query Inputs

**Figure 1:**
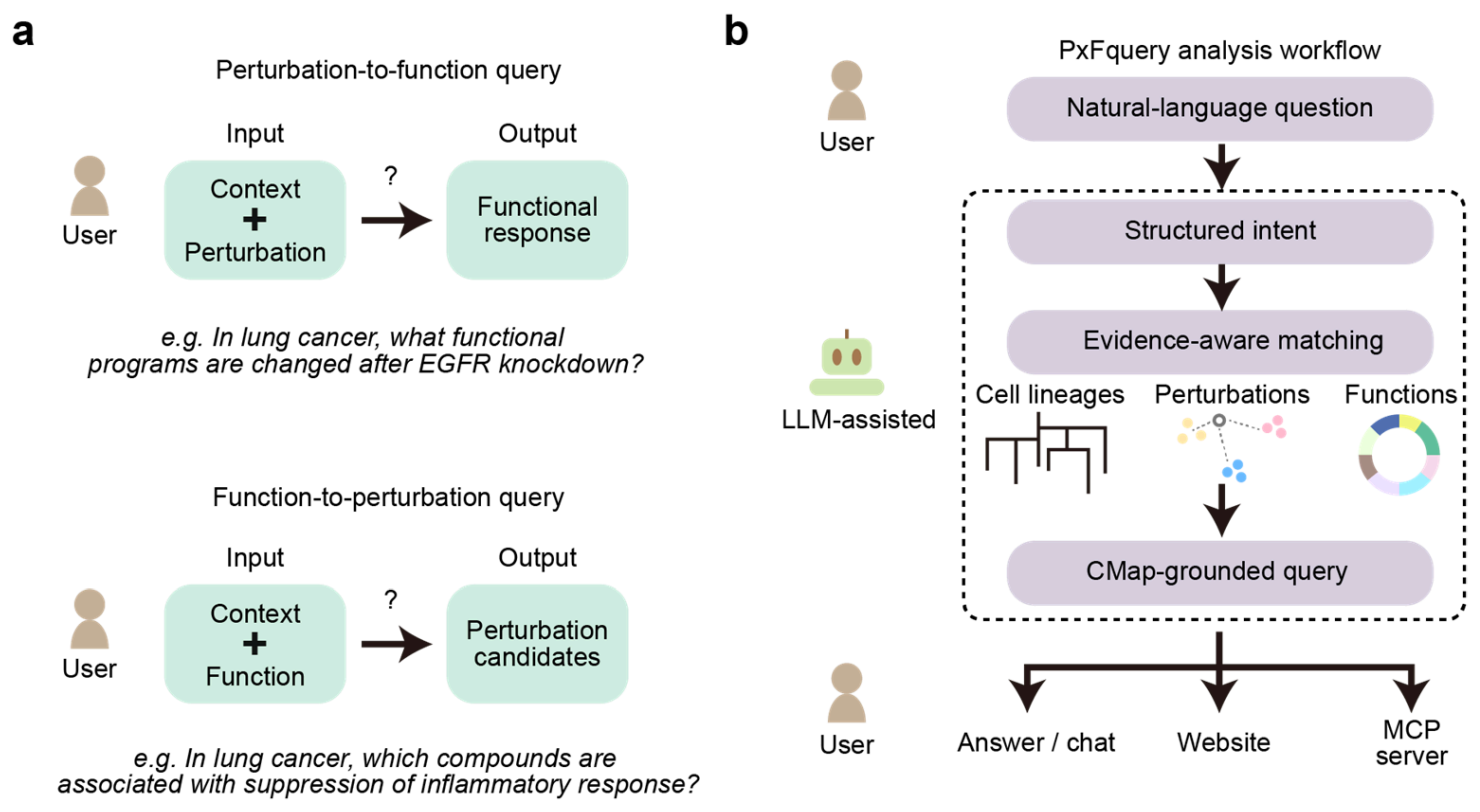
Overview of PxFquery for evidence-grounded perturbation queries. (**a**) PxFquery supports two complementary question forms: which functional response follows a perturbation in a defined cellular context (**top**), and which perturbations match a specified functional state in that context (**bottom**). (**b**) PxFquery analysis workflow. The LLM assists question interpretation, term standardization, evidence assessment, and answer organization, whereas matched indexed CMap/LINCS resources and their precomputed functional scores provide the underlying evidence. The resulting evidence-grounded output can be delivered through multiple presentation interfaces.

PxFquery (v0.5.29) is designed to accept natural-language perturbation query inputs and link them to the precomputed functional score resources described above. An input can start from a cellular context and a given perturbation to ask which functional programs were activated or suppressed by that perturbation. Alternatively, an input can start from a cellular context and a requested functional state to ask which genetic or compound perturbations had functional profiles matching that state. These two input forms correspond to perturbation-to-function and function-to-perturbation query relationships (Figure 1a). PxFquery therefore internally uses multiple LLM prompts to return ranked response evidence and generate a natural-language answer from that evidence (Table S3).

#### 2.3.2. Structured Interpretation and Evidence Matching

During querying, PxFquery loads the functional score matrices, standardized indexes, and observation records from a compressed resource pack, allowing researchers to use the precomputed resources without reprocessing the original CMap/LINCS (Table S4). Researchers can also provide a local resource pack in place of the default resource set. For each natural-language input, an LLM assists the structured interpretation of the cellular context, perturbation or functional target, and query direction. When a phrase does not resolve directly to a single indexed entity, the LLM selects among indexed candidate entities. The workflow from natural-language input to evidence matching and matrix query is shown in Figure 1b.

After term standardization, evidence matching first searches for direct CMap/LINCS measured records in the selected cellular context and perturbation modality. Genetic queries use the specified sh (shRNA) or xpr (CRISPR) modality when provided. Otherwise, both are considered. If no direct measured record is available, PxFquery uses the cell-line hierarchy, compound similarity reference, or gene similarity reference described above to retrieve a limited set of related evidence. Direct and related evidence are then used as the evidence set for functional evidence assembly.

#### 2.3.3. Functional Evidence Assembly

After structured interpretation and evidence matching, PxFquery converts the evidence set into ranked functional evidence. For perturbation-to-function relationships, matched functional score profiles are summarized for the relevant cellular context and perturbation. When multiple signatures supported the same matched relationship, scores are averaged across the supporting signatures. Positive scores are reported in descending order as activated response evidence, and negative scores are reported in ascending order as suppressed response evidence.

For function-to-perturbation relationships, activation and suppression terms from the query are represented as directional functional targets. Candidate genetic or compound perturbations are ranked according to the agreement between their functional profiles and these targets, producing ranked perturbation evidence. This step ranks perturbations represented by measured or related evidence in the precomputed resource. The ranked evidence is assembled into a researcher-readable evidence response that supports natural-language answer generation as well as inspectable tables, visualizations, and reports.

### 2.4. PxFquery’s Implementation and Interfaces

PxFquery was implemented as a Python 3.10 package for running perturbation query analysis in notebooks or scripts. The package follows a scverse/Scanpy-like workflow style for organizing query objects and analysis steps, supporting compatibility with the Python bioinformatics ecosystem [28,29]. Query results can be further rendered as natural-language answers, evidence tables, and figure reports. PxFquery supports configurable LLM providers. In this study, the default LLM configuration uses DeepSeek-V4-Flash for the internal LLM prompts used in the workflow [30,31].

PxFquery also includes a web application at https://caodudu-pxfquery-web.hf.space/ (accessed on 16 July 2026). The website provides a natural-language query entry point and returns evidence-constrained answers and ranked results. Evidence and plot views are used to inspect supporting evidence and its graphical representation. The web application was built with Gradio v5.50.0 and deployed as a Hugging Face Space [32].

PxFquery also provides a Model Context Protocol (MCP) interface for integration with external AI analysis environments [33]. The interface exposes the same evidence-grounded query outputs to MCP-compatible clients during interactive analysis and was tested with OpenCode v1.17.11 [34].

### 2.5. Evaluation Against Experimental Rankings

To evaluate PxFquery against LLM-based approaches, we included two comparators: a direct LLM without external evidence and a PubMed-augmented LLM with retrieved biomedical literature context. The evaluation covered genetic and chemical perturbation-to-function queries, as well as genetic and chemical function-to-perturbation queries. For each evaluation query, directly matching measured records were removed from the evidence set available to PxFquery, so that the comparison tested evidence-guided retrieval beyond direct lookup. The direct LLM received the same query text but did not receive the PxFquery functional score resources or evidence response. The PubMed-augmented LLM comparator received the same natural-language queries with additional PubMed abstract context. PubMed retrieval was performed through the PubMed database, and the retrieved abstracts were provided to the LLM for answer generation.

Reference rankings were established from the complete experimental functional score resources. For perturbation-to-function queries, functional programs were ranked separately in the activated and suppressed directions. For function-to-perturbation queries, candidate perturbations were ranked according to their agreement with the requested functional direction. Each returned item was mapped to the corresponding reference ranking, and the experimental rank percentile was calculated as 100r/N, where r was the 1-based experimental rank and N was the size of the ranking universe. For each query, the median rank percentile of matched top-*k* items was used as the evaluation value, with lower values indicating items closer to the top of the experimental ranking.

Test queries were sampled from experimentally observed CMap/LINCS settings for top-*k* evaluation. We excluded settings where the top-*k* rankings were mainly determined by a limited number of strong signals, with the remaining ranked entries lacking sufficient experimental support. Each selected setting was required to contain at least 10 strongly responding entries (|functional score| > 1) in the corresponding ranking direction. Genetic and chemical perturbation-to-function analyses each used 100 cell-perturbation queries, whereas genetic and chemical function-to-perturbation analyses each used 100 cell-function queries (Table S5). Perturbation-to-function evaluation examined activated and suppressed programs at top 3 and top 5, whereas function-to-perturbation evaluation examined perturbation candidates at top 1, top 3, top 5, and top 10. PxFquery was compared with each LLM-based comparator using paired query identifiers using the Wilcoxon signed-rank test, with tests performed separately for each query family and top-*k* setting.

### 2.6. Evaluation of Natural-Language Query Accessibility

We evaluated the structured interpretation step of PxFquery using BioRED-derived biomedical sentences [35]. BioRED provides multi-round expert manual annotations of biomedical entities. Sentences containing explicit intervention expressions were filtered, resulting in 113 sentences for evaluation. The extracted biological context and perturbation information were compared with the annotations.

We compared PxFquery with an exact keyword metadata query strategy to evaluate accessibility to CMap/LINCS perturbation evidence. The evaluation included 400 cell-perturbation queries derived from PubTator 3.0-annotated publications (200 chemical and 200 genetic queries) [36]. These queries were generated from biomedical research publications and represent perturbation questions arising from real-world biological contexts. Keyword queries were processed using exact matching of cell-line and perturbation metadata, whereas PxFquery used natural-language interpretation and context matching. Retrieval success was defined as obtaining CMap/LINCS perturbation evidence for each query. Retrieval hit rates were compared using one-sided exact McNemar tests.

## 3. Results

### 3.1. Experimental Perturbation Evidence Coverage in PxFquery

**Figure 2:**
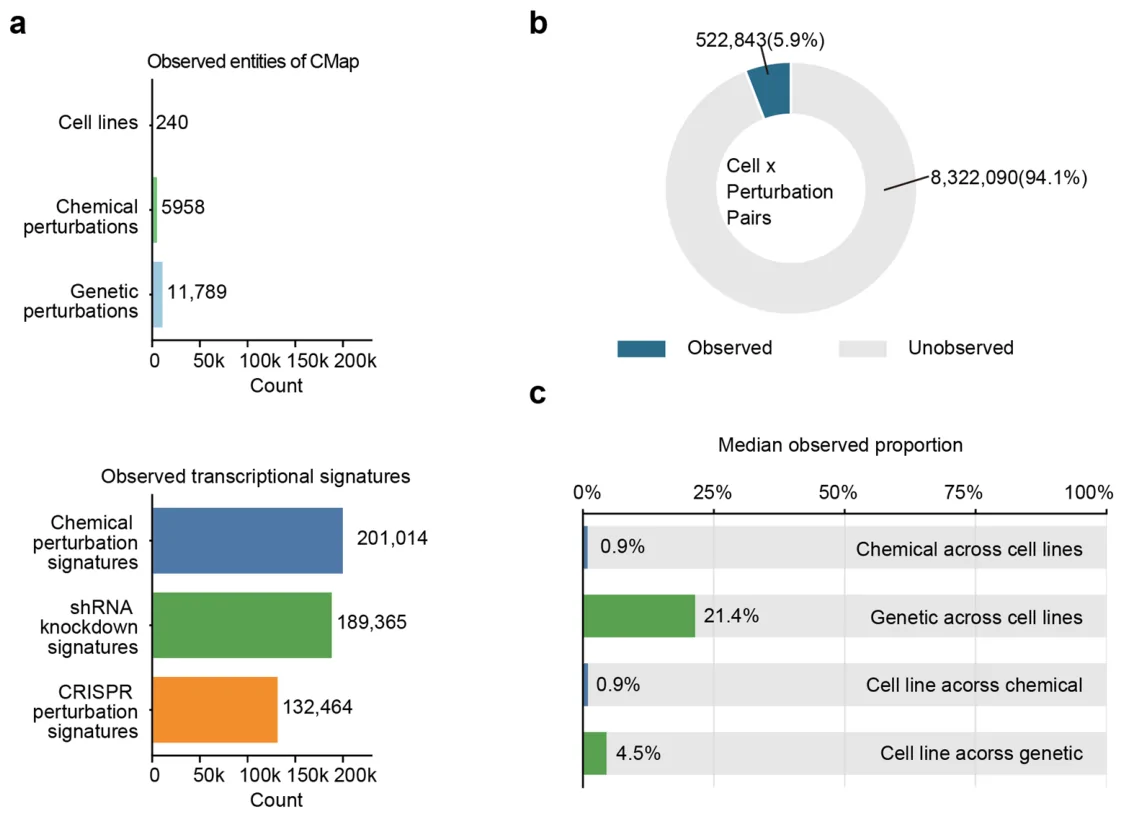
Characterization of perturbation evidence indexed by PxFquery. (**a**) Bar chart showing the scale of the measured perturbation-response resources indexed by PxFquery. (**b**) Direct cell-perturbation coverage of the indexed resource. Donut chart of observed and unobserved pairs across modality-specific grids. Blue: observed pairs. Gray: unobserved pairs. (**c**) Bar chart of median coverage from perturbation-to-cell-line and cell-line-to-perturbation perspectives, separately for chemical and genetic resources.

We first summarized the measured perturbation evidence indexed by PxFquery. The resource encompassed 240 cell lines, 5958 chemical perturbation entities, and 11,789 genetic perturbation entities, together with over 0.2 million chemical perturbation signatures and over 0.3 million genetic perturbation signatures across shRNA and CRISPR modalities (Figure 2a). This large measured response space formed the evidence basis for PxFquery queries.

However, we found that coverage was sparse relative to all potential cell-perturbation combinations. Across the modality-specific grids, only 522,843 pairs were observed, whereas 8,322,090 possible pairs were unobserved, corresponding to 5.9% measured combinations (Figure 2b). This sparsity was present in both query directions: the median coverage of chemical perturbations across cell lines was 0.9%, that of genetic perturbations across cell lines was 21.4%, and the median coverage of cell lines across chemical and genetic perturbations was 0.9% and 4.5%, respectively (Figure 2c and Figure S1). The global cell-line-by-perturbation heatmap further showed that measurements were not distributed uniformly, but instead formed partially offset blocks across cell contexts (Figure S2). Thus, within a sparse large-scale evidence space, unmeasured combinations could still overlap with or approximate existing measurements through cell context or perturbation identity.

**Figure 3:**
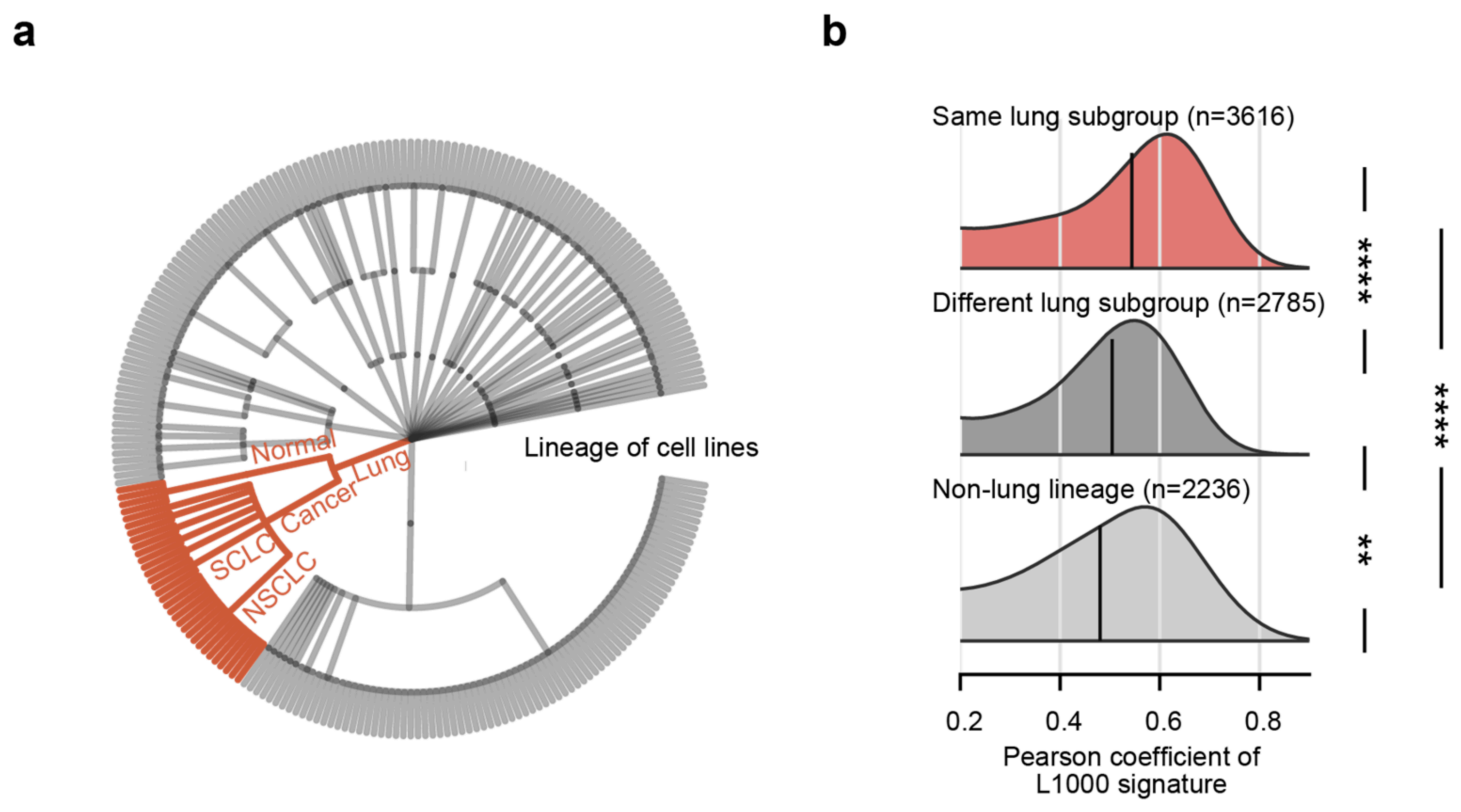
Cell-line lineage relationships and response similarity in PxFquery. (**a**) Circular tree showing cell-line lineage relationships used for related cellular-context matching. The highlighted branch represents lung-related cell lines. (**b**) Pearson correlation distributions of L1000 signatures across cell-line lineage relationships. Groups represent the same lung-cancer subgroup, different lung-cancer subgroups, and lung cancer versus non-lung lineage. *n* indicates the number of evaluated pairs. Statistical test: Kruskal–Wallis with Dunn–Holm correction. Asterisks indicate adjusted *p* values (** *p* < 0.01; **** *p* < 0.0001).

At the level of cellular context, we found that cell-line relationships provided a traceable organizational basis for related evidence. PxFquery used cell-line lineage information to locate a queried cellular context among comparable indexed cell lines (Figure 3a). In the lung-cancer example, related cell lines were delimited by lineage proximity within the lung lineage, the lung-cancer branch, and the small-cell lung cancer (SCLC) or non-small-cell lung-cancer (NSCLC) subbranches. To test whether annotation proximity also corresponded to response proximity, we compared Pearson correlations between L1000 signatures produced by the same perturbation in different lung-related cell lines. Response similarity was highest within the same lung subgroup, followed by different lung subgroups and non-lung lineages (Figure 3b), with significant differences among groups (Kruskal–Wallis test, *p* = 3.70 × 10^−11^; Dunn’s test with Holm correction) (Figure 3b). These observations provided response-level support for using related cell-context evidence when a direct measurement was unavailable.

We assessed the structured interpretation step of PxFquery using BioRED-annotated sentences. PxFquery correctly extracted biological context information in 98 cases (86.7%) and perturbation information in 107 cases (94.7%). We next evaluated whether PxFquery improved access to perturbation evidence compared with exact keyword metadata retrieval. PxFquery achieved higher retrieval rates for both chemical (99.0% vs. 44.5%) and genetic (88.0% vs. 32.0%) perturbation queries (Figure S3; one-sided exact McNemar test, *p* = 1.54 × 10^−33^ and 6.23 × 10^−26^, respectively).

### 3.2. Functional Response Representation in PxFquery

After establishing perturbation evidence and its related cellular contexts, we further organized these responses at the level of functional programs. Tumor development and progression involve a recurring set of core biological activities, allowing perturbation responses to be compressed into a limited set of functional dimensions [37]. To obtain interpretable and comparable dimensions across perturbations, PxFquery integrated MSigDB Hallmark gene sets and 3CA meta-programs into 91 functional programs describing post-perturbation functional responses [6]. We further examined redundancy among these dimensions and found that Jaccard similarities were low for most Hallmark–Hallmark, 3CA MPs–3CA MPs, and Hallmark–3CA MPs pairs (Figure S7). This functional vocabulary therefore retained low redundancy in a limited-dimensional space and provided an interpretive basis for subsequent activated and suppressed functional responses.

**Figure 4:**
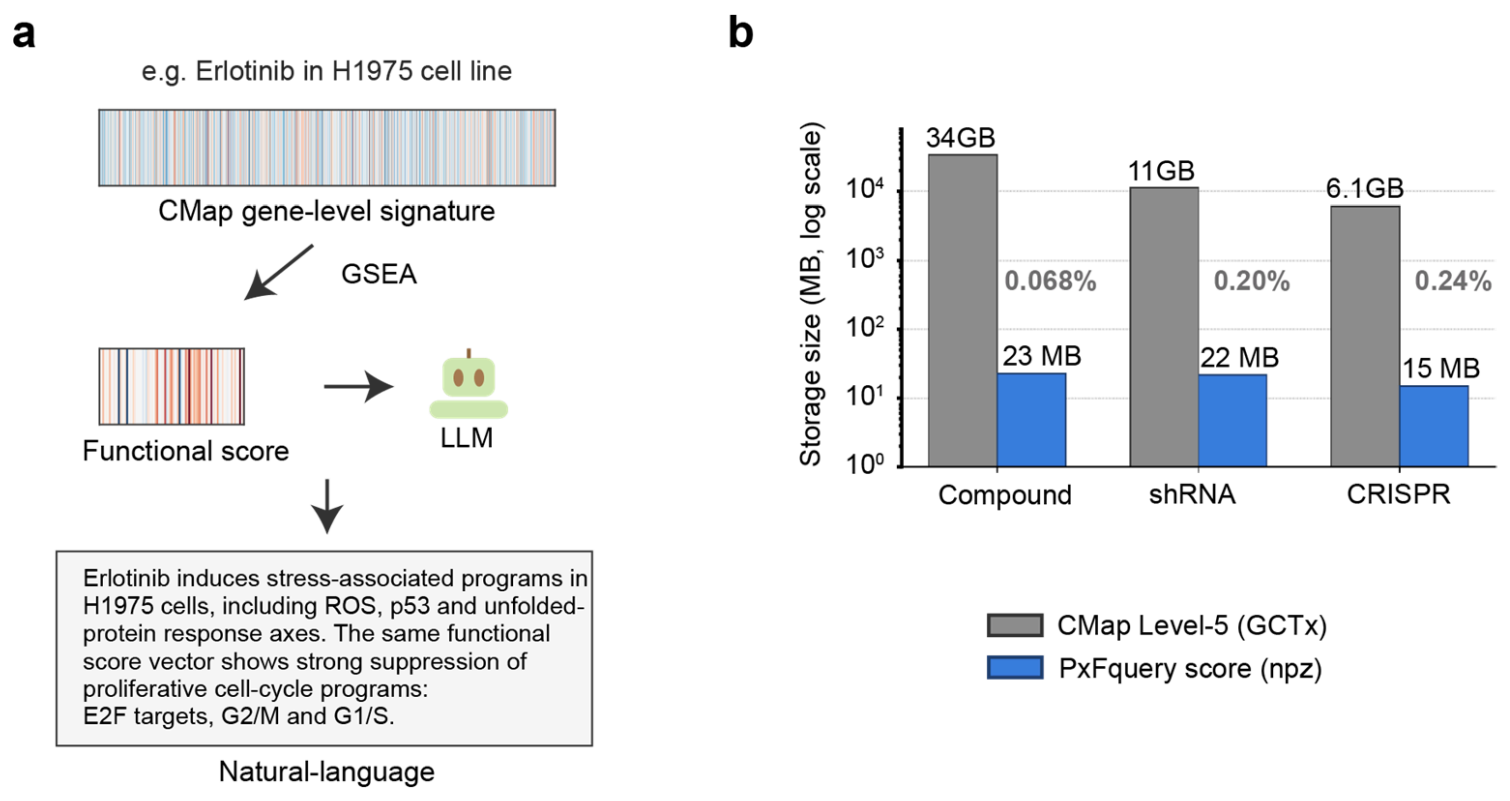
Functional representation of perturbation responses in PxFquery. (**a**) Example conversion of a CMap gene-level signature to a PxFquery functional score for erlotinib in H1975 cells. The functional score is used by the LLM to organize the natural-language result. (**b**) Bar chart comparing the storage size of CMap Level-5 Gene Cluster Text x (GCTx) files and PxFquery score resources in .npz (a NumPy ZIP archive) format. Gray: CMap Level-5 GCTx. Blue: PxFquery score npz. Y axis: log scale.

For erlotinib perturbation in the lung adenocarcinoma cell line H1975, PxFquery compressed 12,328 gene-level features into 91 directional functional scores (Figure 4a). This functional profile retained information on increased or decreased activity after perturbation, including activation of stress-related programs and suppression of cell-cycle programs. The high-dimensional gene signature was thereby converted into directly interpretable functional tendencies. In this functional form, an LLM can directly interpret the post-perturbation functional tendencies and organize an answer around these changes.

This functional compression also markedly reduced the storage required for the evidence resources. When stored in the same 91-program response space, the compound, shRNA, and CRISPR functional-score matrices were reduced from 34 GB, 11 GB, and 6.1 GB to 23 MB, 22 MB, and 15 MB, respectively, corresponding to 0.068%, 0.20%, and 0.24% of their original resources (Figure 4b). We confirmed that these MB-scale matrices could be embedded directly in the PxFquery package for repeated queries.

To evaluate whether the numerical storage optimization affected the functional score matrices, we examined the impact of rounding and precision reduction on score representation. Rounding scores to two decimal places and conversion to float16 largely preserved the relative ranking of functional programs within individual perturbation signatures (Figure S8). Entries set to zero by the $|S_{ij}|<0.1$ threshold showed predominantly high FDR *q* values (Figure S9), indicating that the threshold mainly affected low-confidence scores.

### 3.3. PxFquery-Based Interpretation of Perturbation Responses

**Figure 5:**
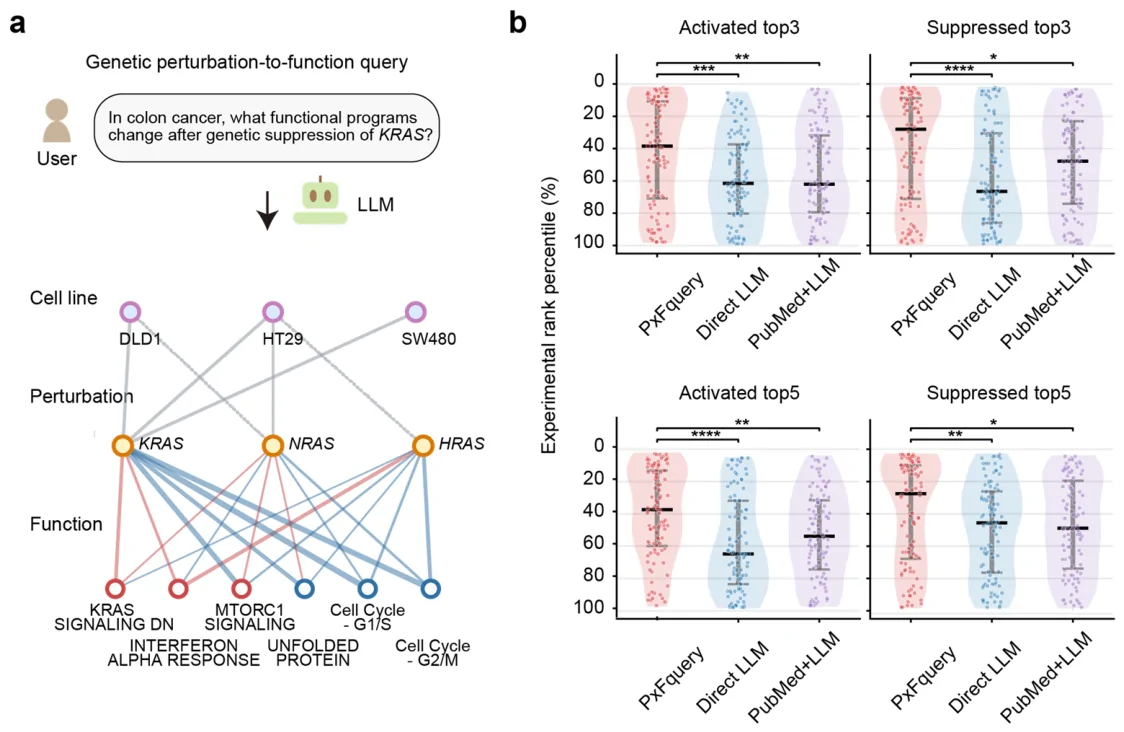
Genetic perturbation-to-function queries and experimental-rank comparison in PxFquery. (**a**) PxFquery result for a genetic perturbation-to-function query of *KRAS* suppression in colon cancer. Red and blue links indicate increased and decreased functional responses, respectively. (**b**) Violin plots of experimental rank percentiles for the top three and top five activated and suppressed functional programs after withholding the exact matching experimental record. Lower percentiles: closer to the top of the experimental reference ranking. Asterisks: * *p* < 0.05; ** *p* < 0.01; *** *p* < 0.001; **** *p* < 0.0001.

Using the experimental data and functional response space in PxFquery, we evaluated the complete workflow from natural-language interpretation to functional explanation. We first applied PxFquery to the question, “In colon cancer, what functional programs change after genetic suppression of *KRAS*?” PxFquery located colon cancer in DLD1, HT29, and SW480 cell lines and integrated *KRAS* loss-of-function experimental evidence from these contexts. The resulting functional interpretation showed increased *KRAS*-suppression-associated functional programs and interferon-related programs, together with reduced G1/S and G2/M cell-cycle programs (Figure 5a and Figure S11a). These functional changes were consistent with reports that *KRAS* inhibition suppresses proliferation in colorectal tumor models [38].

In addition to direct *KRAS* loss-of-function evidence, the query returned related *NRAS* and *HRAS* perturbation evidence with functional responses similar to the *KRAS* evidence. To further analyze related genetic evidence, we visualized the GenePT embeddings used by PxFquery with Uniform Manifold Approximation and Projection (UMAP). *KRAS*, *NRAS*, and *HRAS* clustered closely in this embedding space (Figure S12a). This GenePT embedding neighborhood was consistent with their similar functional responses.

We then tested the agreement between PxFquery-based functional interpretations and experimental rankings across broader genetic perturbation queries. In 100 paired queries with exact matching experimental records withheld, the top three increased functional programs returned by PxFquery had a lower median experimental rank percentile than both direct LLM and PubMed-augmented LLM outputs (Figure 5b). For the top 3 decreased functional programs, PxFquery outputs were also closer to the top of the experimental ranking than both LLM-based comparators. When the comparison was extended to the top 5 functional programs, PxFquery outputs remained closer to the top of the experimental ranking than both direct LLM and PubMed-augmented LLM outputs (Figure 5b).

**Figure 6:**
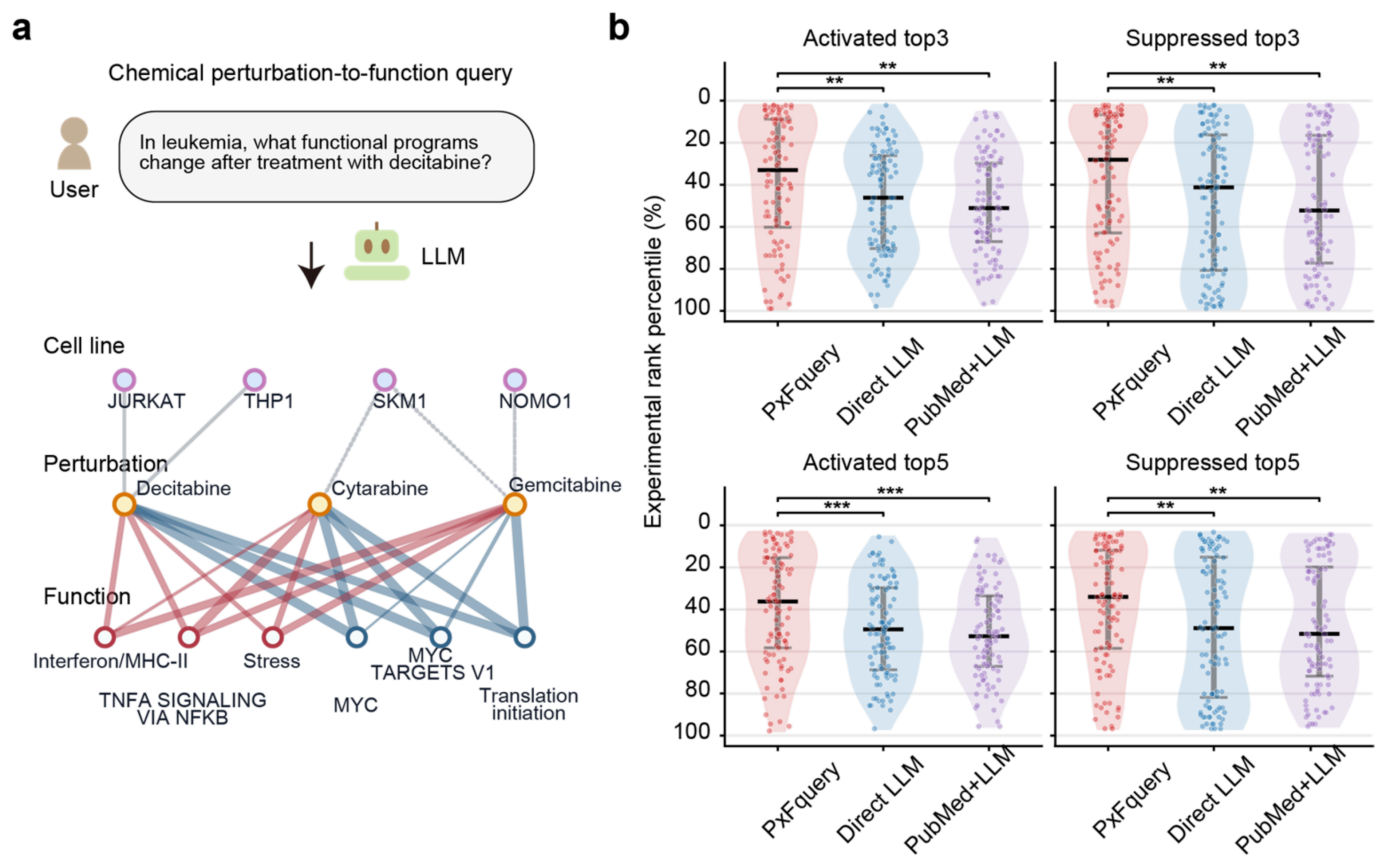
Chemical perturbation-to-function queries and experimental-rank comparison in PxFquery. (**a**) PxFquery result for a chemical perturbation-to-function query of decitabine treatment in leukemia. Red and blue links indicate increased and decreased functional responses, respectively. (**b**) Violin plots of experimental rank percentiles for top 3 and top 5 activated and suppressed functional programs after withholding the exact matching experimental record. Lower percentiles: closer to the top of the experimental reference ranking. Asterisks: ** *p* < 0.01; *** *p* < 0.001.

We next extended the same analysis to chemical perturbation queries. For the question, “In leukemia, what functional programs change after decitabine treatment?”, PxFquery returned a functional interpretation from perturbation evidence across multiple leukemia cell lines (Figure 6a and Figure S11b). The functional changes, including increased interferon-related programs, were consistent with previously reported transcriptional responses to decitabine treatment [39]. To further analyze related compound evidence, we used UMAP to visualize the Tanimoto similarity embedding used by PxFquery and found that decitabine, cytarabine, and gemcitabine occupied the same structural neighborhood (Figure S12b). This structural neighborhood provided a traceable source for the related compound evidence in the query.

Across broader chemical perturbation queries, we further compared experimental rank percentiles across outputs from the three methods. PxFquery outputs showed lower median experimental rank percentiles than both LLM-based comparators for the top three and top five increased or decreased functional programs (paired one-sided Wilcoxon tests; *p* < 0.05; Figure 6b).

To further evaluate PxFquery beyond the CMap/LINCS-derived ranking analysis, we performed an external mechanism-based evaluation using ChEMBL-annotated drug mechanisms [40]. These known drug mechanisms were mapped to drug-functional response relationships for evaluation. We evaluated two relationships: CDK4/6 inhibition-associated E2F suppression and mTOR inhibition-associated mTORC1 suppression. PxFquery showed higher agreement with these externally defined drug-functional response relationships than direct LLM and PubMed-augmented LLM (one-sided exact McNemar tests, *p* < 0.0001; Figure S13).

### 3.4. PxFquery-Based Prioritization of Perturbation Candidates

**Figure 7:**
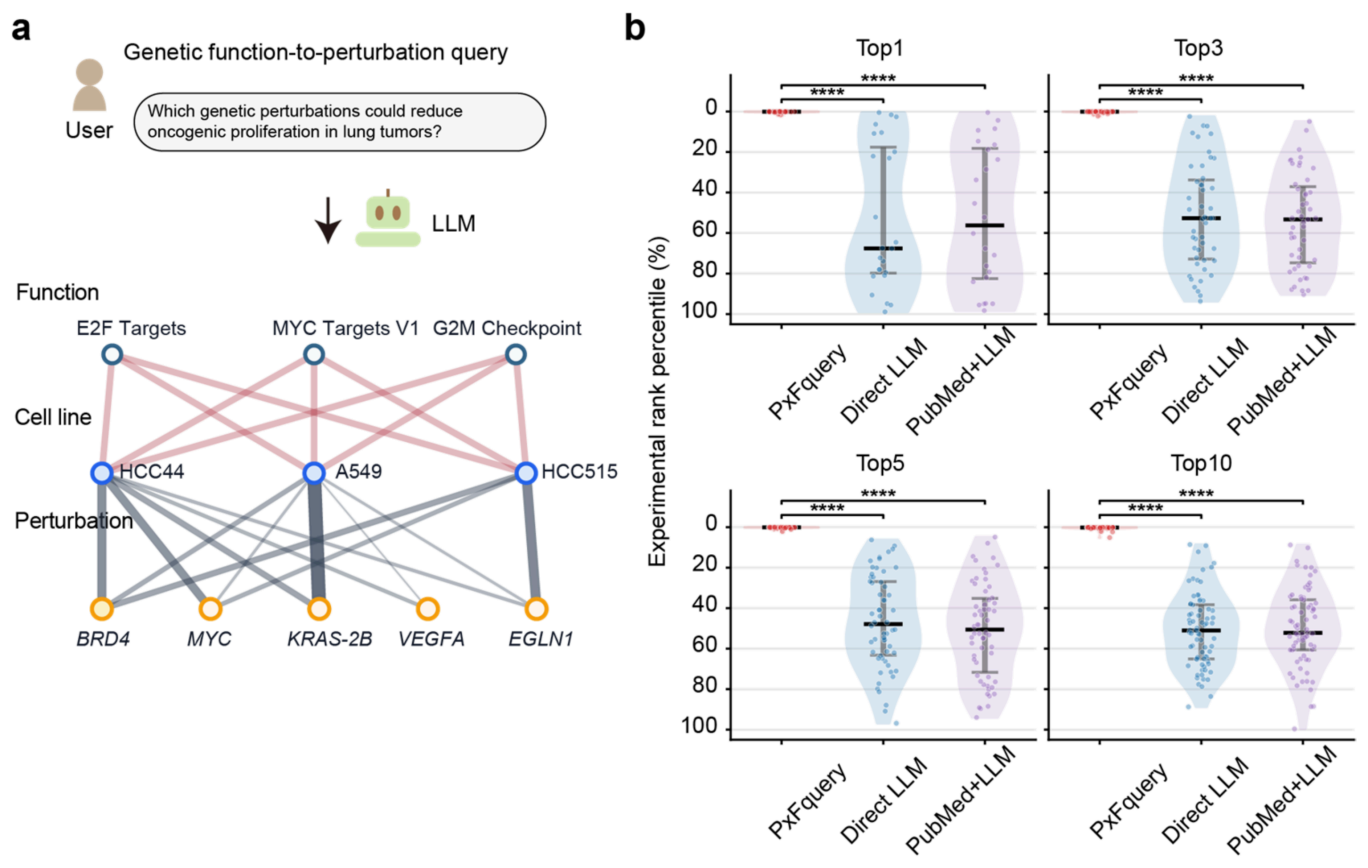
Function-to-perturbation genetic candidate ranking and experimental-rank comparison in PxFquery. (**a**) PxFquery result for a genetic function-to-perturbation query on reducing oncogenic proliferation in lung tumors. (**b**) Violin plots of experimental rank percentiles for top 1, top 3, top 5, and top 10 genetic candidates after withholding the exact matching experimental record. Lower percentiles: closer to the top of the experimental reference ranking. Asterisks: **** *p* < 0.0001.

After interpreting the functional effects of known perturbations, we further examined whether PxFquery returned evidence-supported genetic perturbation candidates from a target functional state. For the question, “Which genetic perturbations may reduce oncogenic proliferation in lung tumors?”, E2F Targets, MYC Targets V1, and G2M Checkpoint defined the target functional state and were linked to lung cancer contexts including HCC44, A549, and HCC515. PxFquery returned *BRD4*, *MYC*, and *KRAS-2B* as the leading genetic candidates, followed by *VEGFA* and *EGLN1* (Figure 7a and Figure S11c). The identification by PxFquery of *BRD4* and *MYC* was consistent with previous reports implicating these factors in proliferative regulation in *KRAS*-mutant NSCLC [41].

To assess the relationship between this candidate ranking and the experimental reference ranking, we further compared candidate ranks across broader genetic function-to-perturbation queries. Before each query, the experimental record that exactly matched the query was removed from PxFquery. From top 1 to top 10, PxFquery candidates had lower median experimental rank percentiles than both direct LLM and PubMed-augmented LLM candidates, with all comparisons being statistically significant (*p* < 0.0001; Figure 7b). These results indicated that the genetic candidate priority order formed from the target functional state was more closely aligned with the experimental reference ranking.

**Figure 8:**
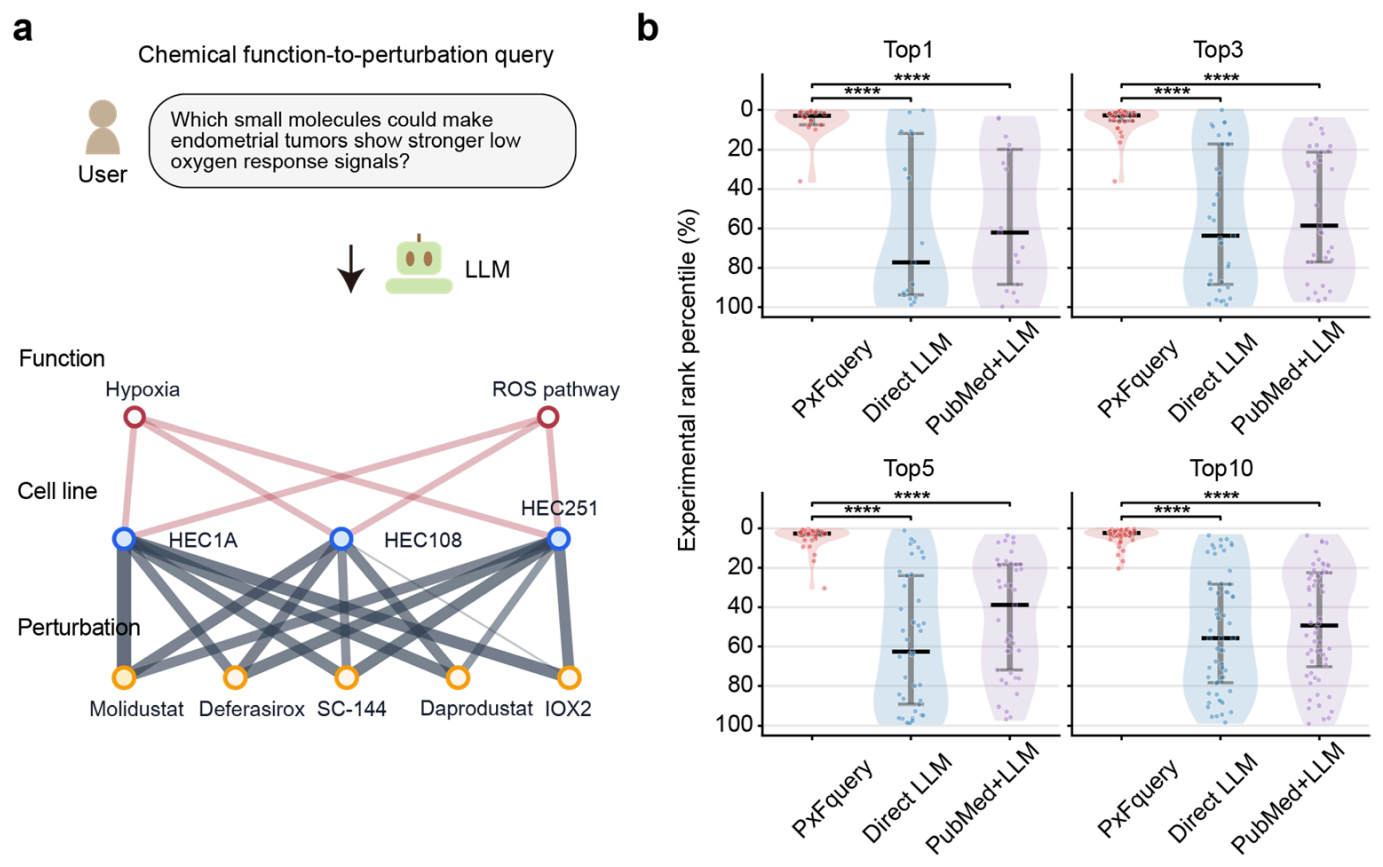
Function-to-perturbation chemical candidate ranking and experimental-rank comparison in PxFquery. (**a**) PxFquery result for a chemical function-to-perturbation query for stronger low-oxygen response signals in endometrial tumors. (**b**) Violin plots of experimental rank percentiles for top 1, top 3, top 5, and top 10 chemical candidates after withholding the exact matching experimental record. Lower percentiles: closer to the top of the experimental reference ranking. Asterisks: **** *p* < 0.0001.

In chemical candidate ranking, we further examined whether the requested functional activation direction was retained. For the question, “Which small molecules may produce a stronger hypoxia response in endometrial tumors?”, the hypoxia and ROS Pathway defined the target functional state. Molidustat, deferasirox, SC-144, daprodustat, and IOX2 were the leading chemical candidates, and their matched evidence corresponded to increased rather than decreased hypoxia-related signaling (Figure 8a and Figure S11d). Molidustat and daprodustat are established HIF-prolyl-hydroxylase inhibitors [42].

Across broader chemical candidate-ranking queries, PxFquery candidates showed lower median experimental rank percentiles than both direct LLM and PubMed-augmented LLM candidates across the evaluated top-*k* settings (*p* < 0.0001; Figure 8b).

### 3.5. Multiple Interfaces of Evidence-Grounded Answering in PxFquery

**Figure 9:**
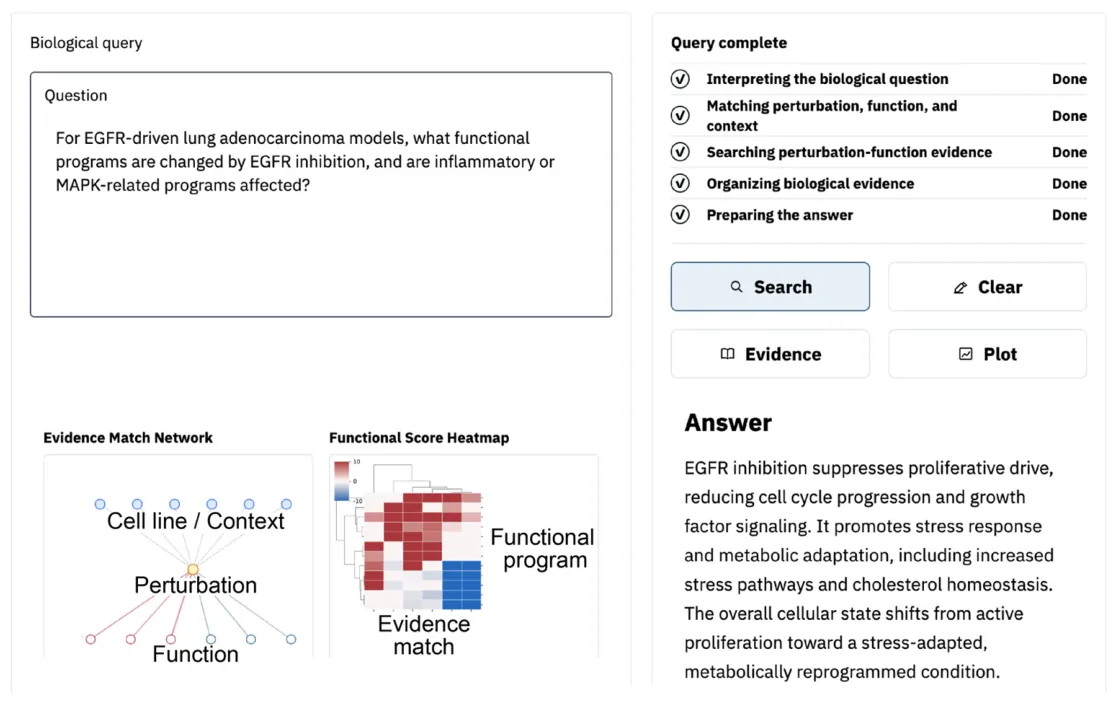
PxFquery web interface for an evidence-grounded perturbation query. Result view for an EGFR-inhibition question in lung adenocarcinoma models.

In addition to the Python package, we developed the PxFquery web interface. Natural-language questions submitted by researchers were parsed into corresponding perturbation evidence queries. The returned result synchronized an LLM-organized natural-language interpretation with the corresponding ranked experimental perturbation evidence (Figure 9). The same query also generated graphical results that further displayed evidence details supporting the natural-language interpretation. Different types of natural-language questions can be completed automatically within the same workflow, without requiring researchers to configure the analysis steps separately.

**Figure 10:**
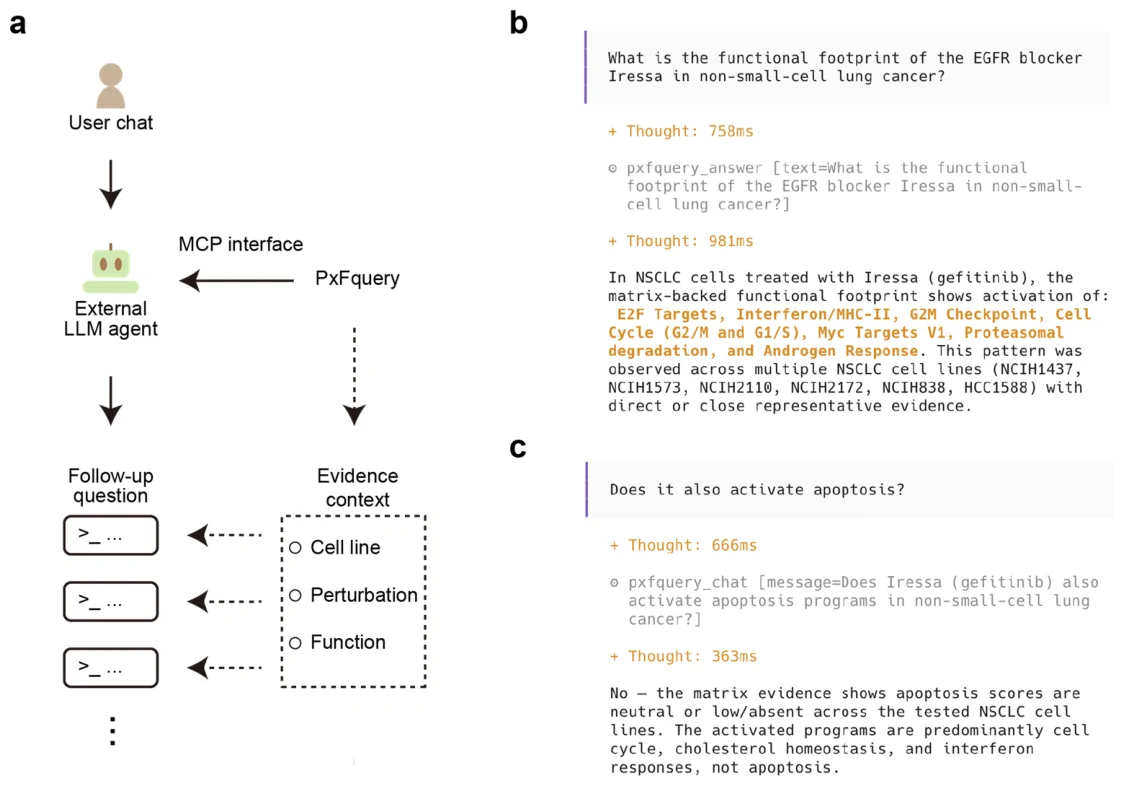
MCP-enabled evidence-context continuity in external AI conversations. (**a**) MCP interface connecting an external LLM agent to PxFquery and retaining cell-line, perturbation, and function context for follow-up questions. (**b**) Example of an initial evidence context for an Iressa perturbation query. (**c**) Example of an evidence-constrained answer to an apoptosis follow-up question.

We further verified PxFquery compatibility with other AI systems. To this end, we developed an MCP mode that enabled PxFquery to connect to MCP-compatible AI products (Figure 10a). We then tested a natural-language perturbation question in OpenCode (Figure 10b). Follow-up questions concerning the same result continued to be answered within the experimental perturbation evidence context established by the initial query.

When a question concerned a function without consistent experimental support, the answer delimited its conclusion accordingly (Figure 10c). To further quantify this evidence-constrained behavior, we evaluated PxFquery using the 200 queries from the genetic and chemical perturbation-to-function evaluations described above. For each query, we generated follow-up questions for the 10 functional programs with the weakest support in the experimental reference ranking. Across all 2000 follow-up questions, PxFquery did not incorrectly interpret these low-support functional programs as supported functional responses (Figure S14). These results support the PxFquery design of providing CMap/LINCS perturbation evidence as experimental context for continuous, evidence-constrained answering of perturbation-related questions.

## 4. Discussion

The PxFquery tool developed in this study brings the analytical use of CMap/LINCS perturbation resources and natural-language queries in perturbation biology into a single workflow. The workflow incorporates an LLM to interpret researchers’ questions and thereby guide the discovery, comparison, and interpretation of perturbation evidence. Compared with conventional query approaches based on keyword or metadata inputs, PxFquery improves access to perturbation evidence by enabling queries directly from biological questions. Compared with direct LLM and PubMed-augmented LLM approaches, PxFquery anchors the query process in measured perturbation evidence from CMap/LINCS. This design allows interpretations and candidate rankings to be traced to specific functional responses and experimental records. Across the evaluated genetic and chemical perturbation-to-function and function-to-perturbation query settings, PxFquery outputs showed closer agreement with experimental reference rankings than the compared LLM-based approaches. Evaluation results demonstrate that integrating large-scale perturbation resources with natural-language interfaces can improve accessibility to experimental evidence and support flexible exploration of perturbation-function relationships.

The embedding-driven similarity-neighborhood index used in this study builds on the recent expansion of representation learning across genes, proteins, and small-molecule drugs [27,43,44]. LangPert, GenePert, and related approaches primarily operate at the gene-signature level by using LLMs or biological representations to model perturbation responses. Functional compression provides a different perspective for utilizing large-scale perturbation resources by reducing high-dimensional gene-level signatures into interpretable functional programs. This lightweight representation enables integration into interactive AI workflows, including MCP-based applications. More importantly, the functional semantic representation supports bidirectional exploration between perturbations and cellular functions. This bidirectional exploration framework facilitates flexible retrieval of perturbation-function evidence and iterative interaction through natural-language follow-up queries. It allows researchers to investigate both the functional consequences of specific perturbations and candidate perturbations associated with desired cellular states. This capability may assist researchers in prioritizing candidate targets and compounds before wet-lab experiments and in constructing validation paths for alternative scientific hypotheses.

Alongside its perturbation-evidence foundation, PxFquery was designed with extensible external knowledge interfaces that can accommodate gene-annotation resources such as GeneCards, drug and chemical resources such as DrugBank and ChEMBL, and PubMed literature records [40,45,46,47]. These interfaces provide additional context for LLM-assisted analysis by linking perturbation evidence with broader bioinformatics knowledge. Future extensions may incorporate additional molecular data modalities, including DNA sequencing data and epigenomic information [48], to further expand the biological context available for perturbation-response analysis. More broadly, the PxFquery workflow is intended to improve the evidentiary rigor and interpretability of perturbation-response analysis, helping transform large-scale perturbation data into knowledge usable for precision-medicine research. Integration with disease-related information via LLM may further support applications such as disease tracking and therapeutic hypothesis generation [49].

Several limitations should be acknowledged. First, CMap/LINCS currently focuses largely on tumor-related cell lines; PxFquery is therefore presently most applicable to tumor-related perturbation questions. We will collect and integrate perturbation data from additional disease settings to broaden this scope. Second, the current tool mainly focuses on single genetic or compound perturbations, while real biological systems may involve more complex perturbation designs and multiple factors that influence response patterns. Future extensions could incorporate combinatorial perturbations, treatment order, and additional biological contexts. Third, the predefined functional space allows comparison across diverse perturbation responses but may not preserve all fine-scale biological details. Researchers can further explore the retrieved evidence records with specific functional terms or downstream analyses for detailed interpretation.

## 5. Conclusions

The use of large-scale perturbation resources remains limited because existing approaches often require structured inputs and specialized bioinformatics knowledge. Improving access to these resources allows researchers to explore perturbation effects directly from biological questions and obtain evidence-supported insights. PxFquery provides a natural-language framework for exploring perturbation-function relationships. The tool supports bidirectional queries between perturbations and functional states and enables lightweight integration into AI workflows. Evaluation showed that PxFquery effectively combines LLM-assisted query interpretation with similarity-based evidence retrieval, with outputs showing closer agreement with experimental reference rankings. Our study shows that natural-language interfaces and large-scale perturbation resources are complementary, supporting AI-assisted response interpretation and perturbation prioritization.

## Data Availability

The data presented in this study are available within the article and its Supplementary Materials. The original CMap/LINCS Level 5 perturbation signatures analyzed in this study are available from the CLUE Data Library (https://clue.io/data, accessed on 24 August 2026). Other publicly available databases and resources used in this study include MSigDB gene sets (https://www.gsea-msigdb.org/gsea/msigdb, accessed on 24 August 2026), Cellosaurus (https://www.cellosaurus.org/, accessed on 24 August 2026), GENCODE (https://www.gencodegenes.org/, accessed on 24 August 2026), PubChem (https://pubchem.ncbi.nlm.nih.gov/, accessed on 24 August 2026), and GenePT (https://github.com/yiqunchen/GenePT, accessed on 24 August 2026). The precomputed PxFquery resource pack is available at https://doi.org/10.5281/zenodo.21001791 (accessed on 24 August 2026) and is automatically downloaded and cached locally by the Python package when used. The PxFquery Python package is available at https://github.com/caodudu/pxfquery (accessed on 24 August 2026). The query sets and structured data underlying the supplementary evaluations are available as JSON files in the PxFquery (v0.5.29) GitHub repository (https://github.com/caodudu/pxfquery/tree/main/supplementary_data, accessed on 24 August 2026). The PxFquery web interface can be accessed and used at https://caodudu-pxfquery-web.hf.space/ (accessed on 24 August 2026). Additional derived data are available from the corresponding author upon reasonable request.

## Supplementary Materials

The following supporting information can be downloaded at https://www.mdpi.com/article/10.3390/genes17091014/s1, Figure S1: Density plots of observed coverage across perturbations and cell lines in the indexed resource. These distributions correspond to the coverage summaries in Figure 2b. Blue: chemical. Green: genetic. Dashed lines: medians; Figure S2: Global coverage of observed cell-line-perturbation pairs in the indexed resource. Heatmap of pair availability across all observed cell lines and perturbations. Blue: observed pairs. Gray: unobserved pairs. Columns: perturbations ordered by cell-line coverage; Figure S3: Retrieval accessibility comparison between keyword metadata queries and PxFquery Bar plots showing retrieval hit rates for chemical (left) and genetic (right) perturbation queries. Gray: keyword metadata queries. Blue: PxFquery. Asterisks indicate significance from one-sided exact McNemar tests (****, *p* < 0.0001); Figure S4: Distribution of NES and functional score values across perturbation signatures. (a) Distribution of normalized enrichment scores (NES) across compound, shRNA, and CRISPR perturbation signatures. (b) Distribution of signed functional scores (S) across compound, shRNA, and CRISPR perturbation signatures; Figure S5: Evaluation of NES and FDR relationships in CMap/LINCS perturbation signatures. (a) Scatter plot showing the relationship between absolute normalized enrichment scores (|NES|) and FDR q values across CMap/LINCS perturbation signatures. (b) Box plots showing the distributions of |NES| (top) and functional scores (bottom) within predefined |NES| bins stratified by FDR significance (*q* ≤ 0.1 and *q* > 0.1); Figure S6: Sensitivity analysis of the *ε* value used in functional score calculation. Distribution of Spearman rank correlations between functional score rankings calculated with alternative *ε* values (10^−8^, 10^−9^, 10^−11^, and 10^−12^) and the default *ε* value (10^−10^); Figure S7: Overlap of gene sets used in PxFqueryDensity plots of pairwise Jaccard similarity among Hallmark gene sets and 3CA MPs (3CA meta-programs). Blue: Hallmark-Hallmark pairs. Purple: 3CA MPs-3CA MPs pairs. Green: Hallmark-3CA MPs pairs. Dashed lines: medians; Figure S8: Rank consistency of functional scores after numerical compression. Distribution of Spearman rank correlations between original and compressed functional score vectors. Left: rounding scores to two decimal places. Right: conversion to half-precision (float16). Correlations were calculated across the 91 functional programs within each perturbation signature; Figure S9: FDR q-value distributions of low-magnitude functional scores. (a) Distribution of FDR q values for functional score entries near the storage threshold (0.095 ≤ |S_ij_| ≤ 0.105). (b) Distribution of FDR q values for functional score entries set to zero during compact storage (|S_ij_| < 0.1); Figure S10: Sensitivity analysis of similarity thresholds in PxFquery. Experimental rank percentiles across different GenePT cosine similarity thresholds (top) and compound Tanimoto similarity thresholds (bottom). Red lines: median experimental rank percentiles. Shaded areas: interquartile ranges. Dotted lines: default similarity thresholds; Figure S11: Four representative query scenarios for PxFquery. (a) PxFquery result for a genetic perturbation-to-function query of KRAS suppression in colon cancer. (b) PxFquery result for a chemical perturbation-to-function query of decitabine treatment in leukemia. (c) PxFquery result for a genetic function-to-perturbation query on reducing oncogenic proliferation in lung tumors. (d) PxFquery result for a chemical function-to-perturbation query for stronger low-oxygen response signals in endometrial tumors; Figure S12: UMAP visualization of preturbations’ neighborhood. (a) GenePT UMAP of the KRAS-family gene neighborhood. Orange: KRAS, NRAS, and HRAS. Gray: all other genes. (b) Tanimoto-similarity UMAP of the decitabine structural neighborhood. Orange: decitabine, cytarabine, and gemcitabine. Gray: all other compound; Figure S13: External mechanism-based evaluation of PxFquery. (a) Comparison of agreement with CDK4/6 inhibition-associated E2F suppression by PxFquery, direct LLM, and PubMed-augmented LLM. (b) Comparison of agreement with mTOR inhibition-associated mTORC1 suppression by the three approaches. Asterisks: one-sided exact McNemar test, **** *p* < 0.0001; Figure S14: Quantitative assessment of evidence-constrained responses in PxFquery. Bar plots showing the percentage of “NO” responses for low-support functional programs in genetic and chemical perturbation queries; Table S1: Perturbation resource coverage used in this study; Table S2: Functional programs and gene membership used in this study; Table S3: Prompt templates used in the PxFquery workflow; Table S4: Runtime resource files used in this study; Table S5: Evaluation details used for experimental-rank comparisons.

## Abbreviations

The following abbreviations are used in this manuscript:

3CA MPs	Curated Cancer Cell Atlas meta-programs
CMap	Connectivity Map
FDR	false discovery rate
GCTx	Gene Cluster Text x
GSEA	gene set enrichment analysis
LINCS	Library of Integrated Network-based Cellular Signatures
LLM	large language model
MCP	Model Context Protocol
MSigDB	Molecular Signatures Database
NES	normalized enrichment score
NSCLC	non-small-cell lung cancer
ORA	over-representation analysis
SCLC	small-cell lung cancer
UMAP	Uniform Manifold Approximation and Projection
