# Supplementary material for: PxFquery: A Bioinformatics Tool for Large Language Model-Assisted Functional Analysis of Large-Scale Perturbation Signatures
Source: Genes (Basel). 2026 Aug 27;17(9):1014. doi: 10.3390/genes17091014 (PMC13606428; doi:10.3390/genes17091014)
Supplement: Supplementary file 1 [file genes-17-01014-s001.zip › Supplementary Figure.pdf]

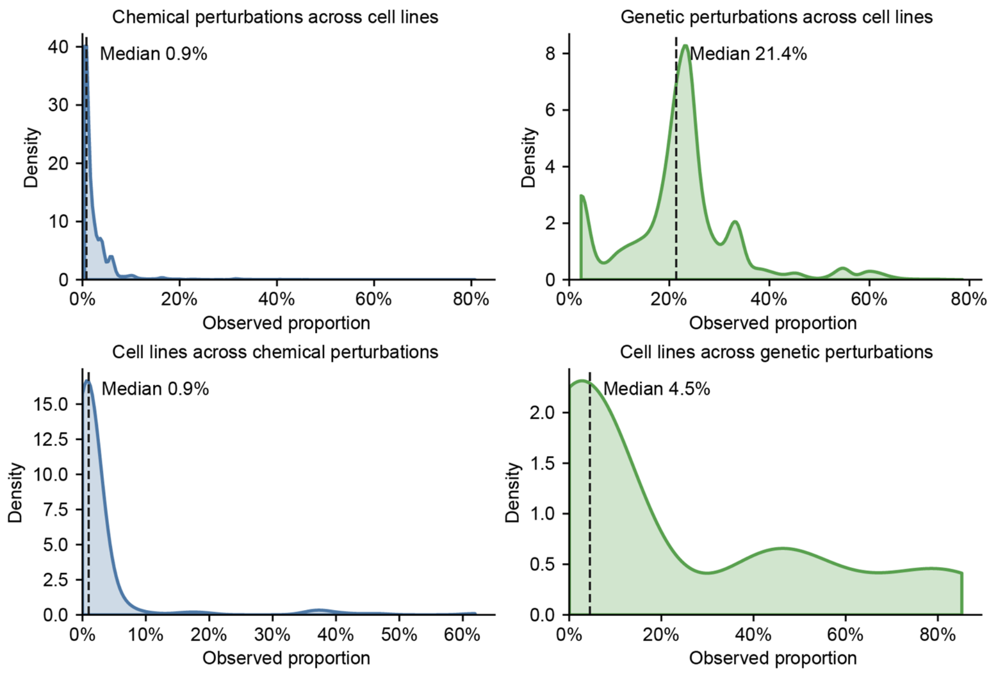

Figure S1. Density plots of observed coverage across perturbations and cell lines in the indexed resource. These distributions correspond to the coverage summaries in Figure 2b. Blue: chemical. Green: genetic. Dashed lines: medians.

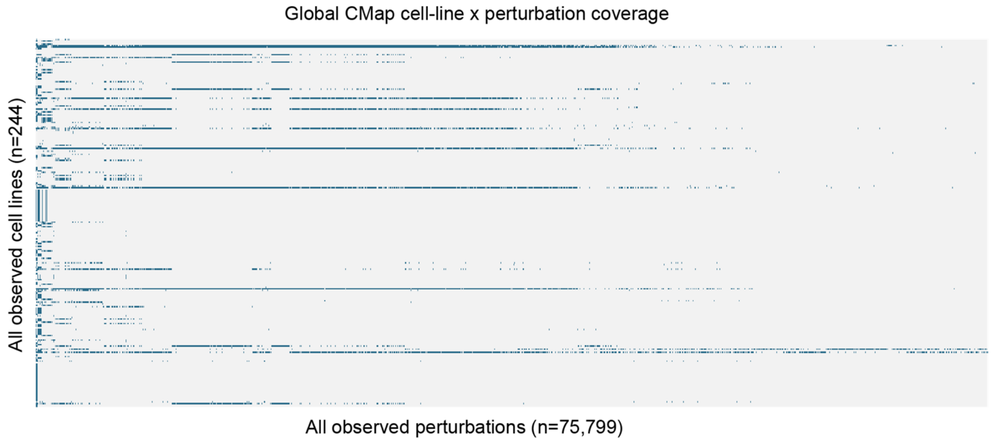

Figure S2. Global coverage of observed cell-line-perturbation pairs in the indexed resource. Heatmap of pair availability across all observed cell lines and perturbations. Blue: observed pairs. Gray: unobserved pairs. Columns: perturbations ordered by cell-line coverage.

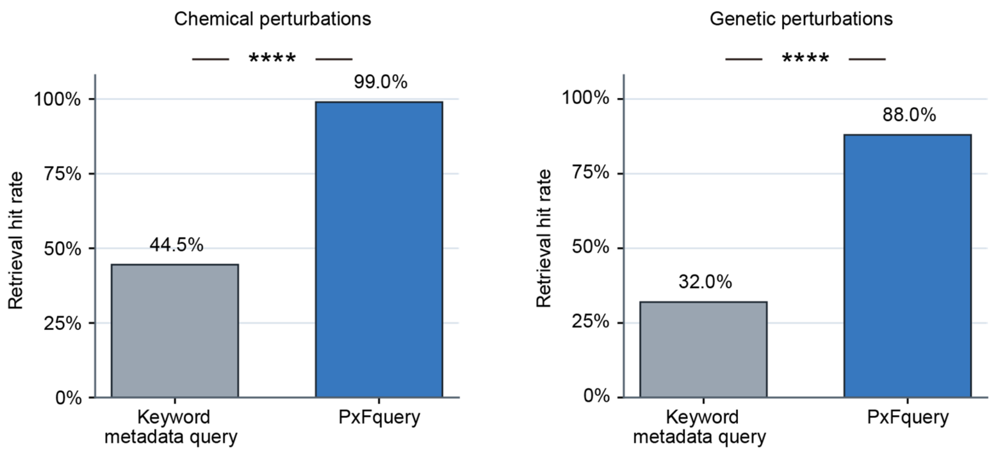

Figure S3. Retrieval accessibility comparison between keyword metadata queries and PxFquery. Bar plots showing retrieval hit rates for chemical (left) and genetic (right) perturbation queries. Gray: keyword metadata queries. Blue: PxFquery. Asterisks indicate significance from one-sided exact McNemar tests (****, $p < 0.0001$).

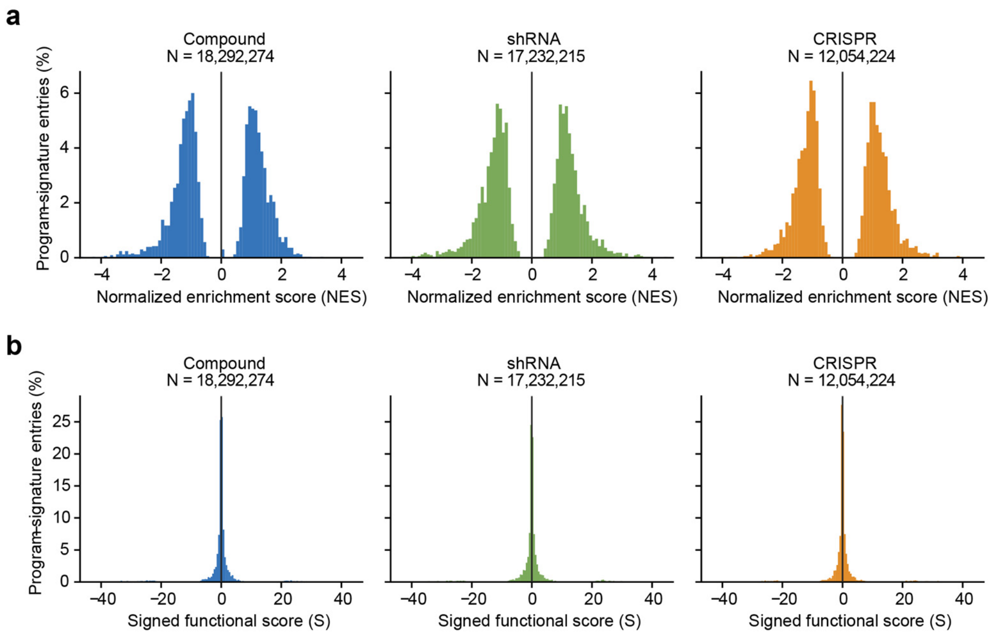

Figure S4. Distribution of NES and functional score values across perturbation signatures. (a) Distribution of normalized enrichment scores (NES) across compound, shRNA, and CRISPR perturbation signatures. (b) Distribution of signed functional scores (S) across compound, shRNA, and CRISPR perturbation signatures.

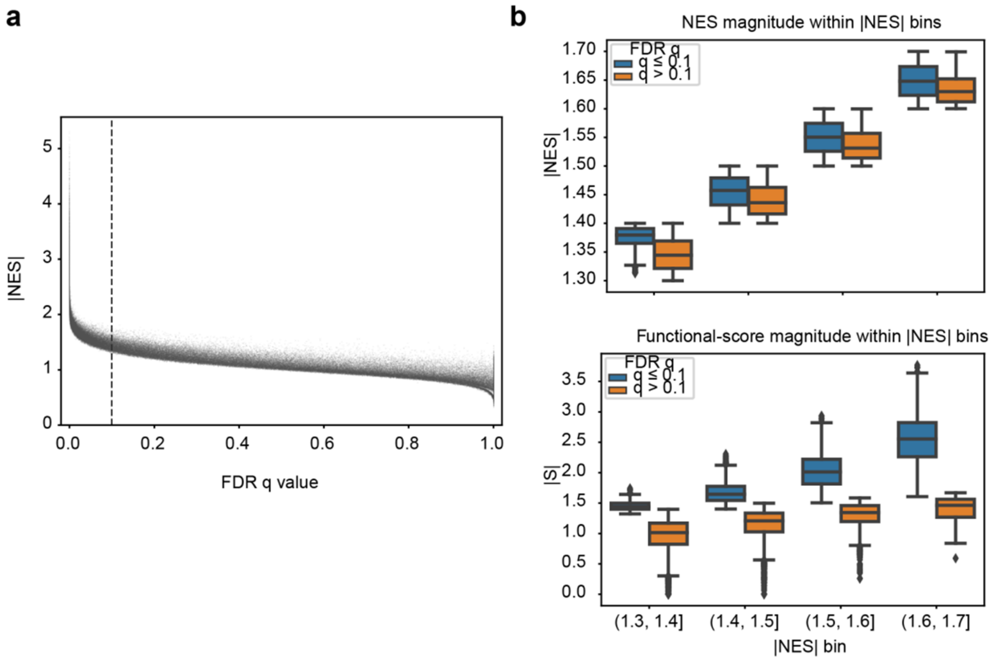

Figure S5. Evaluation of NES and FDR relationships in CMap/LINCS perturbation signatures. (a) Scatter plot showing the relationship between absolute normalized enrichment scores (|NES|) and FDR q values across CMap/LINCS perturbation signatures. (b) Box plots showing the distributions of |NES| (top) and functional scores (bottom) within predefined |NES| bins stratified by FDR significance ($q \leqslant 0.1$ and $q > 0.1$).

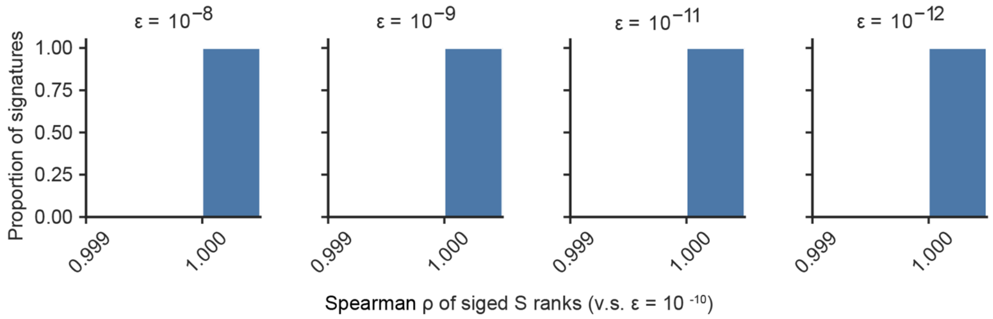

Figure S6. Sensitivity analysis of the ε value used in functional score calculation. Distribution of Spearman rank correlations between functional score rankings calculated with alternative ε values (10^{-8}, 10^{-9}, 10^{-11}, and 10^{-12}) and the default ε value (10^{-10}).

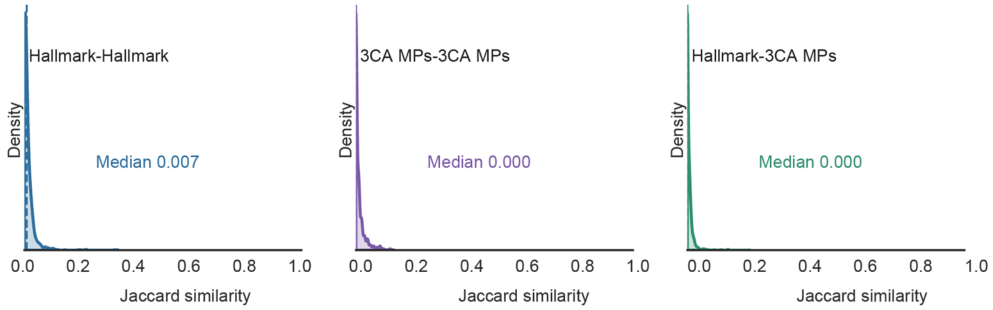

Figure S7. Overlap of gene sets used in PxFquery. Density plots of pairwise Jaccard similarity among Hallmark gene sets and 3CA MPs (3CA meta-programs). Blue: Hallmark-Hallmark pairs. Purple: 3CA MPs-3CA MPs pairs. Green: Hallmark-3CA MPs pairs. Dashed lines: medians.

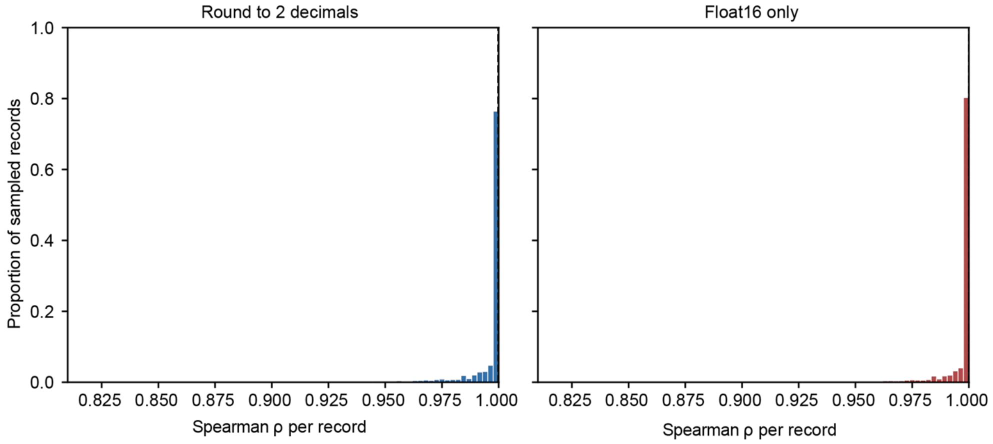

Figure S8. Rank consistency of functional scores after numerical compression. Distribution of Spearman rank correlations between original and compressed functional score vectors. Left: rounding scores to two decimal places. Right: conversion to half-precision (float16). Correlations were calculated across the 91 functional programs within each perturbation signature.

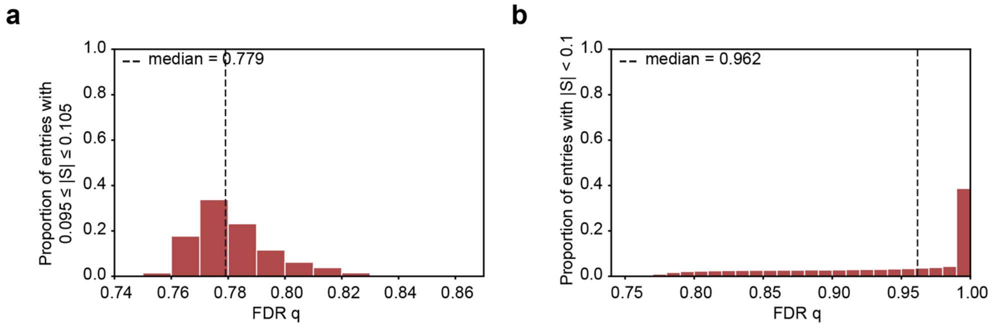

Figure S9. FDR q-value distributions of low-magnitude functional scores. (a) Distribution of FDR q values for functional score entries near the storage threshold ($0.095 \leqslant |S_{ij}| \leqslant 0.105$). (b) Distribution of FDR q values for functional score entries set to zero during compact storage ($|S_{ij}|<0.1$).

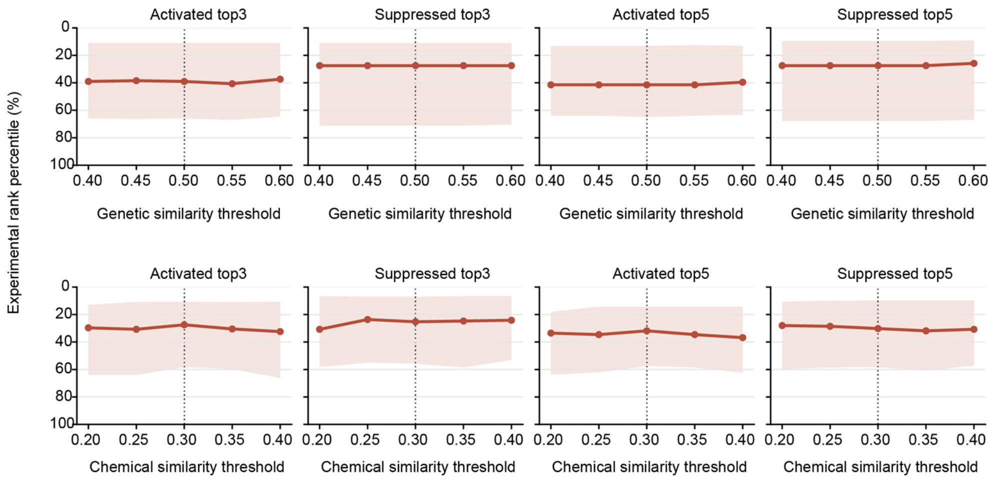

Figure S10. Sensitivity analysis of similarity thresholds in PxFquery. Experimental rank percentiles across different GenePT cosine similarity thresholds (top) and compound Tanimoto similarity thresholds (bottom). Red lines: median experimental rank percentiles. Shaded areas: interquartile ranges. Dotted lines: default similarity thresholds.

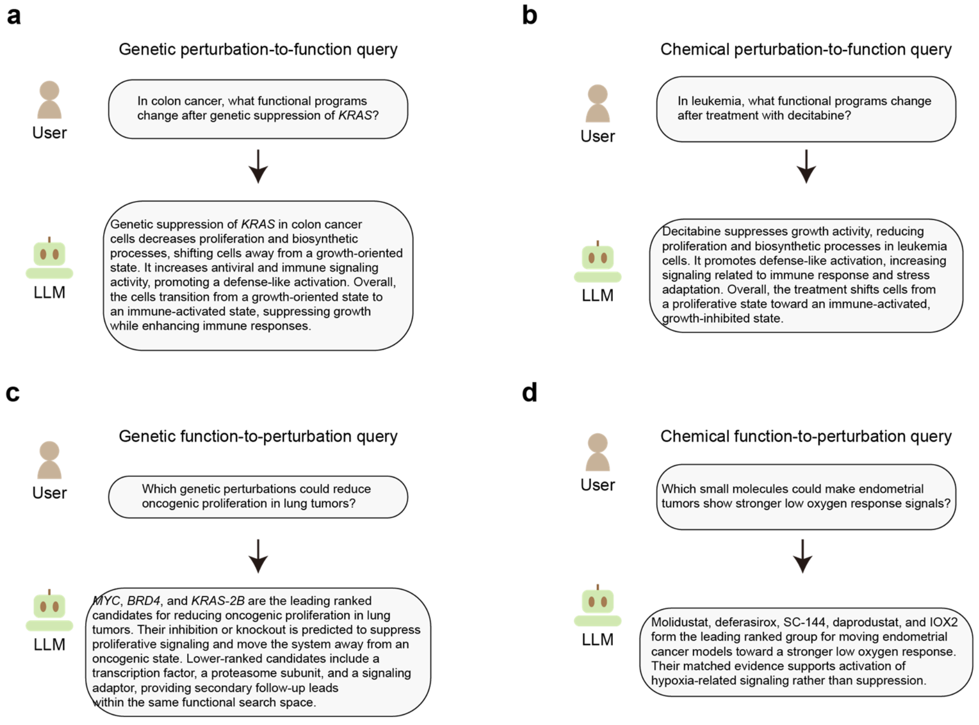

Figure S11. Four representative query scenarios for PxFquery. (a) PxFquery result for a genetic perturbation-to-function query of KRAS suppression in colon cancer. (b) PxFquery result for a chemical perturbation-to-function query of decitabine treatment in leukemia. (c) PxFquery result for a genetic function-to-perturbation query on reducing oncogenic proliferation in lung tumors. (d) PxFquery result for a chemical function-to-perturbation query for stronger low-oxygen response signals in endometrial tumors.

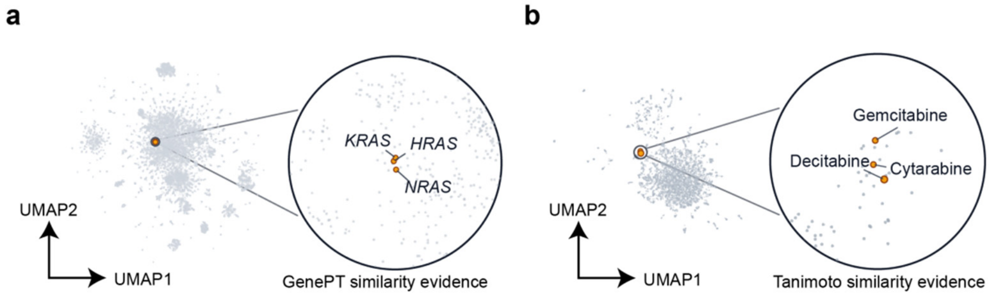

Figure S12. UMAP visualization of preturbations' neighborhood. (a) GenePT UMAP of the *KRAS*-family gene neighborhood. Orange: *KRAS*, *NRAS*, and *HRAS*. Gray: all other genes. (b) Tanimoto-similarity UMAP of the decitabine structural neighborhood. Orange: decitabine, cytarabine, and gemcitabine. Gray: all other compounds.

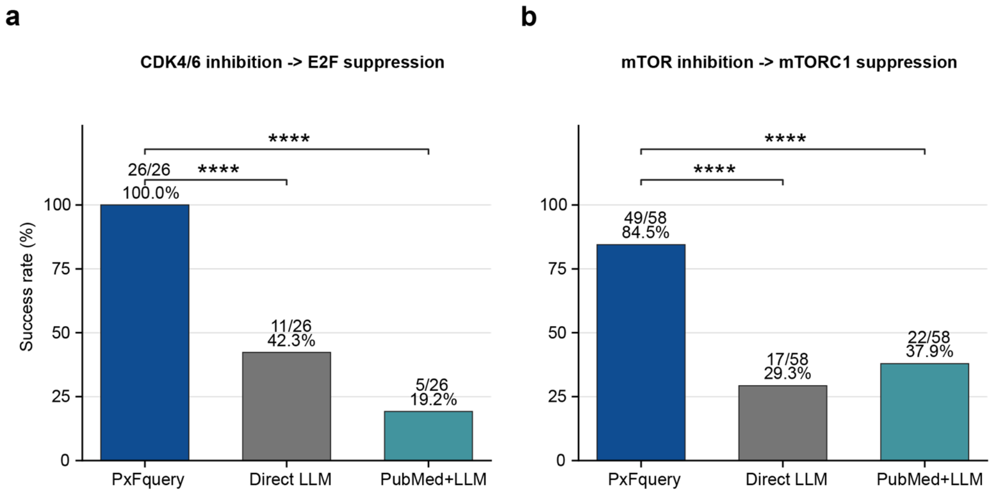

Figure S13. External mechanism-based evaluation of PxFquery. (a) Comparison of agreement with CDK4/6 inhibition-associated E2F suppression by PxFquery, direct LLM, and PubMed-augmented LLM. (b) Comparison of agreement with mTOR inhibition-associated mTORC1 suppression by the three approaches. Asterisks: one-sided exact McNemar test, **** $p < 0.0001$.

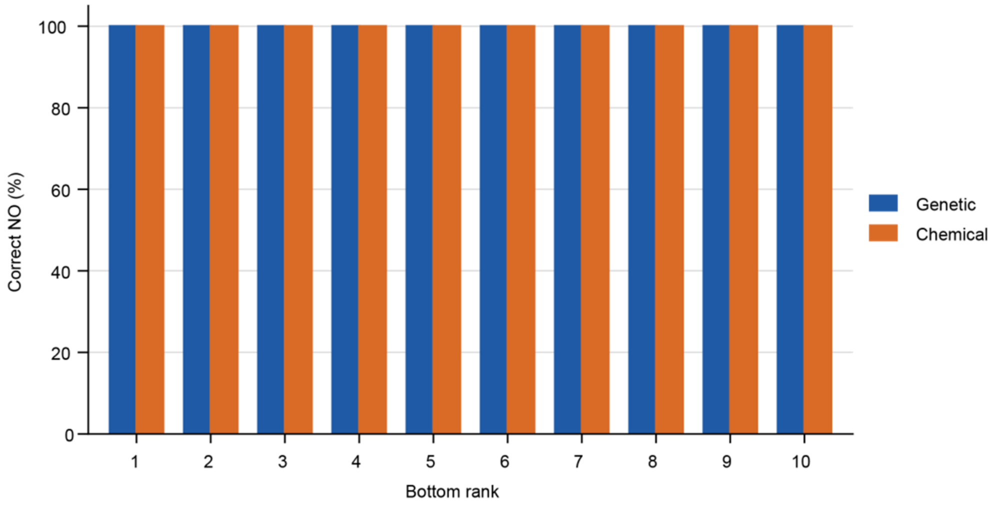

Figure S14. Quantitative assessment of evidence-constrained responses in PxFquery. Bar plots showing the percentage of “NO” responses for low-support functional programs in genetic and chemical perturbation queries.
